# Supervised Analysis for Phenotype Identification: The Case of Heart Failure Ejection Fraction Class

**DOI:** 10.3390/bioengineering8060085

**Published:** 2021-06-21

**Authors:** Cristina Lopez, Jose Luis Holgado, Raquel Cortes, Inma Sauri, Antonio Fernandez, Jose Miguel Calderon, Julio Nuñez, Josep Redon

**Affiliations:** 1 Cardiovascular and Renal Research Group, INCLIVA Research Institute, University of Valencia, 46010 Valencia, Spain; crislozu@incliva.es (C.L.); josholsan@gmail.com (J.L.H.); Raquel.Cortes@uv.es (R.C.); isauri@incliva.es (I.S.); afernandez@incliva.es (A.F.); jmcalderon@incliva.es (J.M.C.); 2 Cardiology Hospital Clínico of Valencia, 46010 Valencia, Spain; yulnunez@gmail.com; 3 Internal Medicine Hospital Clínico of Valencia, 46010 Valencia, Spain; 4 CIBERObn, Carlos III Health Institute, 28029 Madrid, Spain

**Keywords:** heart failure, phenotype, left ventricular ejection fraction, primary care, artificial intelligence, supervised analysis

## Abstract

Artificial Intelligence is creating a paradigm shift in health care, with phenotyping patients through clustering techniques being one of the areas of interest. Objective: To develop a predictive model to classify heart failure (HF) patients according to their left ventricular ejection fraction (LVEF), by using available data from Electronic Health Records (EHR). Subjects and methods: 2854 subjects over 25 years old with a diagnosis of HF and LVEF, measured by echocardiography, were selected to develop an algorithm to predict patients with reduced EF using supervised analysis. The performance of the developed algorithm was tested in heart failure patients from Primary Care. To select the most influentual variables, the LASSO algorithm setting was used, and to tackle the issue of one class exceeding the other one by a large amount, we used the Synthetic Minority Oversampling Technique (SMOTE). Finally, Random Forest (RF) and XGBoost models were constructed. Results: The full XGBoost model obtained the maximum accuracy, a high negative predictive value, and the highest positive predictive value. Gender, age, unstable angina, atrial fibrillation and acute myocardial infarct are the variables that most influence EF value. Applied in the EHR dataset, with a total of 25,594 patients with an ICD-code of HF and no regular follow-up in cardiology clinics, 6170 (21.1%) were identified as pertaining to the reduced EF group. Conclusion: The obtained algorithm was able to identify a number of HF patients with reduced ejection fraction, who could benefit from a protocol with a strong possibility of success. Furthermore, the methodology can be used for studies using data extracted from the Electronic Health Records.

## 1. Introduction

Artificial intelligence (AI), an interdisciplinary science with multiple approaches, is a wide-ranging branch of computer science. Advancements in machine learning and deep learning are creating a paradigm shift in virtually every sector, including medicine, with phenotyping patients through clustering techniques being one of the areas of interest [1]. The goal of phenotyping patients is to allow for the identification of patient subgroups with similar presentation, prognosis and response to therapy. 

Heart failure (HF) is a major health care problem worldwide, for which left ventricular ejection fraction (LVEF) has established clinically useful phenotypes for guiding treatment to reduce associated mortality and morbidity [2,3]. Classically, with heart failure, there are two recognized LVEF phenotypes: reduced LVEF (HFrEF) and preserved (HFpEF) [4,5]. However, recently, The European Society of Cardiology added a third intermediate LVEF phenotype. Although ejection fraction (EF) class is an important predictor of the treatment response data available in electronic health records (EHR), there is frequently a lack of EF quantitative values, limiting their usefulness in clinical and health service research [6]. An immediate next step is to develop algorithms and strategies and identify their distinct phenotypes in the absence of EF measured by echocardiography.

When looking for a proxy to identify HF phenotypes, AI methods could be useful in combining information that is usually collected in EHRs. Machine learning is an application of artificial intelligence, which focuses on how computers learn from data whose methods and techniques are increasingly applied in medicine. Disease identification [7] and pathology and image diagnosis [8,9], as well as clinical research, are some of the main applications of machine learning in epidemiology and clinical medicine, among others [10]. To explore phenotypes of patients with chronic HF, prior studies have already used hierarchical clusters to classify HFpEF patients [11,12]. These studies are mainly focused on the definition of phenotypes rather than predicting the EF class by using a proxy that makes the decision based on available information from the EHR. 

In this current study, we developed a predictive model that classifies HF patients according to their LVEF by using available features such as age, gender and present diseases. The goal was to overcome the performance of previous studies by using a new approach, supervised analysis, a subfield of machine learning where models can be trained to predict the class of the target variable with earlier knowledge of the output values from prior data [13]. 

## 2. Materials and Methods

### 2.1. Data Source and Study Population

**Figure 1:**
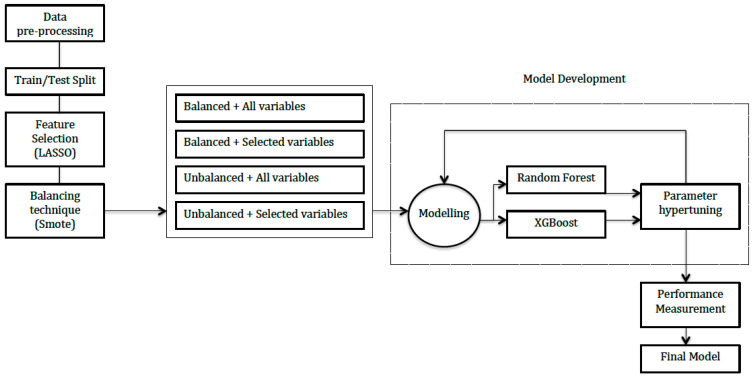
Full process diagram.

Subjects older than 25 years with a diagnosis of heart failure, ICD-9 codes (402.X1, 404.X1,404.X3,428 and 398.91) were selected from the EHR system of a community of people over 25 years in 2012 (Supplementary Material, Figure S1). From this database, we selected a group with available LVEF values measured by echocardiography classified as HFrEF and HFpEF according to the EF measurement, LVEF < 40% and ≥ 40%, respectively. A second group of patients with an HF diagnosis in the absence of LVEF values in the EHR, was collected with or without regular follow-up by Cardiology Departments (Figure 1). The variables to be tested were selected from those codified in the ICD-9. The study was approved by the Ethical Committee of the Hospital Clinico of Valencia in the scope of the BigData@Better Heart, a project founded in the IMI2 program (IMI2-FPP116074-2). Consent forms were obtained from the patients who took part in the echocardiography study and the data of the second group were documented by a process of pseudo-anonymization, making it impossible to use this information to identify the patients, since the only link between the data and the patient is a code not available to the researchers.

### 2.2. Selection of Variables and Analytical Procedure

Variables included demographic information, age and gender and ICD-9-codified diseases. The original dataset was split into two different partitions, corresponding to 80% and 20% of the original dataset. The first, a training set, was used to train the different models developed in the study. The second, a test set, was used to measure the performance of the models developed with the training set. This partition was performed using the *caret* package in R. This allows us to split our data by maintaining the proportion of classes in both partitions. The most influential variables were considered using feature selection methods.

### 2.3. Feature Selection

The least absolute shrinkage and selection operator (LASSO) was used. This method creates a regression model, where the estimated coefficients β_i_ for each variable suffer a penalization [14,15] or are set to zero. In the following equation, we can see the general formula of the regression model expressed in vector notation

Y = Xβ + ε(1)

where Y is the end-point vector (our target), X is the vector of the covariates in our model, β is the vector of the coefficients for these covariates, and ε is a random error.

The estimation of β parameters is typically performed by minimizing the sum of squares of the residuals; this is called the Ordinary Least Squares (*OLS*) approach, and the loss function being minimized is the following:

(2) $$\;L_{OLS}\left(\hat{\beta }\right)=\overset{n}{\underset{i=1}{\sum }}{\left(y_{i}-{x^{\prime }}_{i}\hat{\beta }\right)}^{2}$$

When LASSO is used, the LASSO penalization term is added to this formula, resulting in the following equation:

(3) $$L_{OLS}\left(\hat{\beta }\right)=\overset{n}{\underset{i=1}{\sum }}{\left(y_{i}-{x^{\prime }}_{i}\hat{\beta }\right)}^{2}+\lambda \overset{m}{\underset{j=1}{\sum }}\left|{\hat{\beta }}_{j}\right|$$

Here, λ is known as the regularization penalty. Say λ is set to zero, then:

(4) $$L_{OLS}{}^{\prime }\left(\hat{\beta }\right)=\overset{n}{\underset{i=1}{\sum }}{\left(y_{i}-{x^{\prime }}_{i}\hat{\beta }\right)}^{2}=L_{OLS}\left(\hat{\beta }\right)$$

then, minimizing $L_{OLS}{}^{\prime }\left(\hat{\beta }\right)$ means minimizing $L_{OLS}\left(\hat{\beta }\right)$. Otherwise, if λ is set to 1, Equation (3) turns into:

(5) $$L_{OLS}{}^{\prime }\left(\hat{\beta }\right)=\overset{n}{\underset{i=1}{\sum }}{\left(y_{i}-{x^{\prime }}_{i}\hat{\beta }\right)}^{2}+\overset{m}{\underset{j=1}{\sum }}\left|{\hat{\beta }}_{j}\right|=L_{OLS}\left(\hat{\beta }\right)+\overset{m}{\underset{j=1}{\sum }}\left|{\hat{\beta }}_{j}\right|$$

and minimizing $L_{OLS}{}^{\prime }\left(\hat{\beta }\right)$ means minimizing $\overset{m}{\underset{j=1}{\sum }}\left|{\hat{\beta }}_{j}\right|$, which makes the value coefficients much lower than in the λ = 0 case. To choose an optimum λ value, we defined a set of λ values, and for each λ, we estimated $\hat{\beta }$ such that $L_{OLS}{}^{\prime }\left(\hat{\beta }\right)$ is minimal. Then, we had two paired sets of λ and $\hat{\beta }$ values. Those covariates that are not set to zero are usually the final ones selected.

Additionally, when features agree, the complexity of the model is reduced. We performed a LASSO algorithm, setting the EF value as the endpoint ($y_{i})\;$and introducing the rest of the covariables to the model $\left({x^{\prime }}_{i}\right)$.

### 2.4. Imbalanced Data Distribution

To tackle the issue of one class exceeding the other one by a large proportion, we used the Synthetic Minority Oversampling Technique (SMOTE) [16,17] included in the *DMwR* package. This algorithm creates new minority class examples by extrapolating between existing ones. Although matching seems to be a convenient procedure to perform before building any classification model, we created a predicting model using the original database. In order to avoid the problem of data leakage, the different techniques applied over the data, such as SMOTE, feature selection and hyperparameter tuning, should only be applied in the training set, not in the test set.

### 2.5. Model Development

We created several models based on two different machine learning algorithms, Random Forest (RF) and XGBoost [18], to compare their overall performance. We constructed reduced RF and XGBoost models with four possible previous algorithm performances in the dataset: balanced data in combination with feature selection (LASSO) or using all of the variables; unbalanced data, in combination with either feature selection or all of the variables. Then, we defined a set of values for the model hyperparameters. A grid was used, where the following hyperparameters were introduced: (a) Random Forest: number of threes and number of candidate variables at each split; (b) XGboost: subsample ratio, ratio of subsample columns by tree, maximum tree depth, learning rate, regularization terms and partition threshold. We used k = 5 cross validation in the training set to test all possible combinations and find the most convenient tuning.

### 2.6. Tools Used for Preparing and Running the Models

R 4.1.0 software (R Foundation for Statistical Computing, Vienna, Austria) packages was used in all of the processes that follows:

- Database partition caret package;
- Balancing the dataset DMwR package;
- LASSO implemented using glmnet package;
- Train with Random Forest and XGBoost packages;
- Plots ggplot2;
- Performance metrics Caret, ROCR & PRROC packages.

### 2.7. Performance Measurements

In each model, several performance measurements were calculated, optimizing sensitivity and specificity measurements, but also taking Negative Predictive Value (NPV), Positive Predictive Value (PPV) and Accuracy into account. We made this choice because we aimed to isolate the HFrEF class. In this way, we could be sure that those predicted to be HFrEF (or Positive) would truly be HFrEF.

When comparing between models with similar sensitivity and specificity values, we selected the best by focusing on the other metrics and the Precision–Recall Curve (PR Curve), since it gives a more informative picture of the model’s performance than the ROC Curve when the datasets are highly skewed [19,20].

A diagram showing the full process is displayed in Figure 1.

## 3. Results

### 3.1. Characteristics of the Study Population

A total of 2854 subjects with HF diagnoses and LVEF measurement were included. Mean age was 74 years old and 47% were females. Diabetes was present in 53.4% of the participants and hypertension was present in 82.3% of the participants, the highest percentage of all the comobidities. In the complete dataset, HFrEF was present in 23.4%. A total of 2284 patients were used to train the models, while 570 were used to test. This partition maintains the proportion of HFrEF and HFpEF registers of the complete dataset. From the variables contained in the EHR, 13 appear to have been candidates for the models. Age and sex distribution, as well as the prevalence of the relevant variables in the study population, are shown in Table 1.

### 3.2. Models Developed

**Figure 2:**
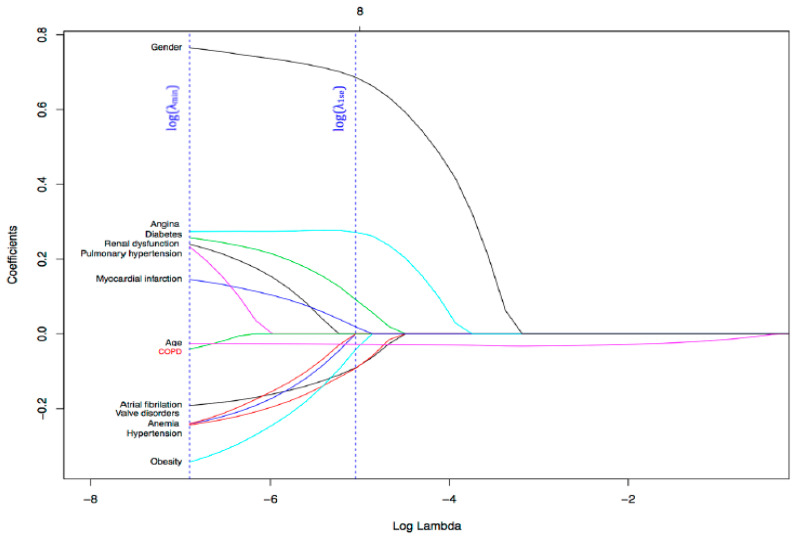
The most relevant coefficients of the model based on their contribution in the EF class prediction. On the *x*-axis, Log Lambda refers to the logarithmic of the regularization parameter (λ), which controls the weight that each variable has in the model.

We constructed two types of models: reduced and full. To obtain reduced models, we defined a set of different λ values. For each λ value, the algorithm built a single model, adding the penalization (λ) to the coefficients. The most relevant variables are those that appeared in many models, which means that the associated coefficients were non-zero. Gender, age, unstable angina, atrial fibrillation (AF) and acute myocardial infarct (AMI) were the variables that most influence EF value. In Figure 2, the *x*-axis represents logarithmic lambda values (λ).

**Figure 3:**
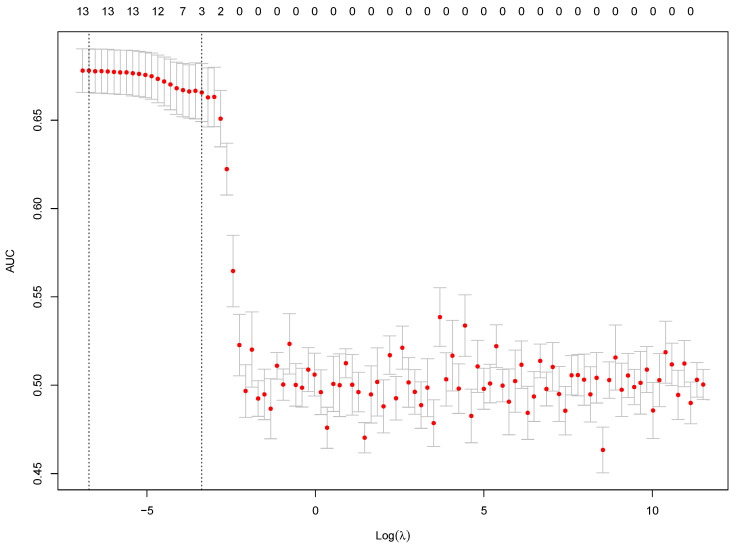
Using the training set, AUC values and its 95% confidence intervals for different logarithmic values of λ represented on *x*-axis, where λ is the regularization parameter in the model. Vertical lines correspond to Log(λ_min_) and Log(λ_se_) which are the most optimal Log(λ) values.

**Figure 4:**
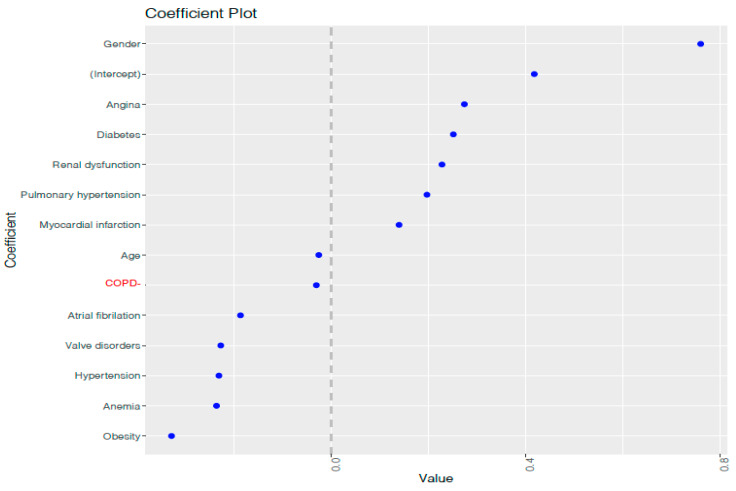
Coefficients’ values fixing λ = λ_min_ in the LASSO model. The up and down positions on the *y*-axis show the variables which most contribute to the model as its associated coefficients take the highest values in the training set. On the middle positions, the variables which contribute less to the model are located, as its associated coefficients are close to 0.

To define the most convenient λ value, the λ that maximizes the area under the curve (AUC) was selected. In Figure 3, the two vertical lines indicate two optimum log(λ) values: the first one from the left corresponds to log (λ_min_), the value that maximizes the AUC model’s, while the second corresponds to log (λ_se_). Afterwards, we built a model setting λ at λ_min_, and the variables whose coefficients were greater or lower than 0 were the predictor variables selected for the final model. The coefficient values associated with each variable: age (−0.02), gender (0.76), atrial fibrillation (−0.18), angina (0.27), hypertension (−0.23), valve disorders (−0.22), diabetes (0.25), anemia (−0.23), COPD (−0.03), pulmonary hypertension (0.19), obesity (−0.32), renal dysfunction (0.22) and myocardial infarction (0.14), Figure 4. These values represent the contribution of each covariate to the endpoint, with the highest value having the highest importance.

After the feature selection process, we constructed two new datasets: the first one (Balance 1) resulted from oversampling the minority class in the original dataset until it reached the majority class size. In the second one (Balance 2), the minority class was oversampled, maintaining a reasonable balance between both classes without equalizing their sizes. Original and new dataset sizes and proportions are shown in Table 2.

Table 3 summarizes the performance of the candidate models. All the results were obtained from testing these models in the testing dataset. While NPV was high among all models (ranging between 0.84 and 0.88), PPV presented a higher variance (0.44 as the lowest value vs. 0.75 as the highest). Note that the models performed with the original dataset reached a higher accuracy, varying from 0.80 to 0.84, as compared to models performed with balanced datasets. C-statistics were around 0,70 and the Precision–Recall Curve (AUCpr) obtained the highest values with the original datasets, with its maximum value obtained with the Random Forest model (0.51).

### 3.3. Models Performance

The full model from the original dataset, obtained with XGBoost, was applied in a large dataset of 79,057 HF patients, and among them, 26,376 patients were treated in primary care in the absence of a routine cardiology consultation and without available LVEF, Table 4. Applying the algorithm can identify patients with HFrEF: 19060 among all patients with HF and 6359 among those without a regular cardiology consultation.

## 4. Discussion

Left Ventricle Ejection Fraction phenotypes guide the management of HF patients, but are frequently not recorded in the EHR from primary care. Having alternatives to help estimate the HF class could be helpful, not only for research in health care services but also for physicians to choose better treatment. In the present study, a machine learning algorithm to predict the phenotype category based on the main characteristics and diseases of the patient was developed. The full XGBoost model excelled because it offered better modelling, maximized the sensitivity, and reached a high NPV. This present approach could be applied to other clinical conditions.

Few studies attempted to develop methods to predict left ventricular ejection fraction in patients with heart failure. Some used administrative claims from Medicare [21,22,23] or a specific database such as the Swedish Heart Failure Registry [24]. For those using administrative claims, a large number of variables were used in the training sample, identified by the ICD-code. On the other hand, the Swedish Heart Failure used a restricted number of variables but included laboratory parameters and treatments. The studies differ from this present one in that this study includes EF-measured patients and uses a different methodological approach. Lee et al. [23] identified atrial fibrillation, obesity, pulmonary, hypertension and valvular disease as being significantly associated with the development of heart failure with HFpEF, while male gender, history of cardiomyopathy, and myocardial infarction were significantly associated with the risk of heart failure with HFrEF. Overall, and despite limitations, routine clinical characteristics could potentially be used to identify different EF subphenotypes in databases.

Previous studies have also developed statistical and unsupervised learning algorithms to classify LVEF phenotypes. In the Desai study [22], the analysis included 11,073 patients, which was much larger than our sample size. Furthermore, the proportion of HFrEF and HFpEF individuals was well-balanced, leading to an easier distinction between classes. Despite the above, the overall accuracy of the selected binomial logistic model did not overcome the measures that we obtained with our final model. Our analysis was based on supervised analysis and, although machine learning techniques are far from being emergent technologies, its application on LVEF measure prediction is certainly innovative. In this study, we used SMOTE, LASSO and two powerful algorithms: Random Forest and XGBoost. Synthetic minority oversampling technique (SMOTE) is one of the most commonly used oversampling methods to solve imbalance problems in the training and test datasets. It aims to balance class distribution by randomly increasing minority class examples by replicating them. Although the concept is very promising when stuck with extremely skewed data, it does not always improve the model results, such as in our case. However, in this kind of study, it can be very useful.

Continuing onto a brief description of these algorithms, Random Forest is a combination of Decision Tree algorithms and Bagging, where both belong to supervised analysis. Together, they train a model to predict the class of the target variable with earlier knowledge of the output values deduced from prior data [13]. The other technique used, XGBoost, combines Boosting and Gradient Boosting algorithms. Boosting sequentially the corrects the errors committed by the previous models, which wrongly classified the elements, while Gradient Boosting tries to modelize the residuals, that is, transform the errors into a function to avoid overfitting [13]. We chose these algorithms because these were the most suitable for the dataset and also for the binary class of the target. In addition, they achieved the best results among the other models, based on alternative machine learning algorithms, such as Naive Bayes, Support Vector Machine and Artificial Neural Network. In particular, XGBoost is becoming popular in machine-learning competitors and data scientists, as it has been battle tested for production on large-scale problems [20].

Applying the algorithm to the large amount of data for patients with HF allowed around 24% of patients with HFrEF who would benefit from more precise treatment to be recognized. Future research will include time variables such as time-to-inclusion from diagnoses dates and medication and hospital admissions. Furthermore, exploring other balancing techniques, such as generating synthetic data based on the individual characteristic distribution, could lead to analytical improvement.

There are some limitations to our research which should be mentioned. As we collected the information from the EHR system, there was not a large number of patient’s LVEF measures and, furthermore, we dealt with an unbalanced dataset, as HFrEF represents a minority of the total HF patients. In addition, there was a wide variety of performance measurements that could be used to evaluate the models. Depending on the characteristics and the goal of the problem, some metrics will perform better than others. The selection of the optimum λ was based on the AUC metric, which is the most intuitive and typically used metric. Finally, our goal was to maximize the PPV value which entails a relative lower value in the sensitivity analysis.

Heart Failure Guidelines stratify patients based on the LVEF in HFrEF or HFpEF [4,25], In those with HFrEF, well-defined treatment strategies improve the risk of hospitalization and survival and, therefore, a clear treatment algorithm is recommended. In those with HFpEF, no treatment has demonstrated an improvement in the outcomes to date. Identifying patients with HFrEF in the absence of measured LEVF can help to introduce treatments which have been successful. In addition, it can help to retrieve real world data from large databases in epidemiological, health care burden and cost studies.

In conclusion, the presented step-by-step AI approach, in the case of the HF phenotype, is a methodology that can help to obtain phenotypes from partially completed databases for different diseases, a common scenario in the EHRs.

## Figures and Tables

**Table 1:** Demographics and chronic diseases tested.

Variable	Training Dataset			Test Dataset		
	HFrEF	HFpEF	Total	HFrEF	HFpEF	Total
	n = 535	n = 1749	n = 2284	n = 133	n = 437	n = 570
Demographics						
Male	386 (72.15)	832 (47.57)	1218 (53.33)	92 (69.17)	203 (46.45)	295 (51.75)
Age, mean (SD)	71.85 (11.14)	75.8 (9.89)	74.88 (10.33)	69.92 (10.62)	75.6 (9.8)	74.27 (10.27)
Comorbidities						
Atrial fibrillation	197 (36.82)	776 (44.37)	973 (42.6)	32 (24.06)	225 (51.49)	257 (45.09)
Anemia	171 (31.96)	745 (42.6)	916 (40.11)	45 (33.83)	199 (45.54)	244 (42.81)
Diabetes	317 (59.25)	911 (52.09)	1228 (53.77)	71 (53.38)	224 (51.26)	295 (51.75)
Hypertension	418 (78.13)	1470 (84.05)	1888 (82.66)	93 (69.92)	368 (84.21)	461 (80.88)
Obesity	49 (9.16)	226 (12.92)	275 (12.04)	11 (8.27)	72 (16.48)	83 (14.56)
Pulmonary HTN	26 (4.86)	71 (4.06)	97 (4.25)	3 (2.26)	22 (5.03)	25 (4.39)
CKD	88 (16.45)	245 (14.01)	333 (14.58)	12 (9.02)	69 (15.79)	81 (14.21)
Valve disorders	66 (12.34)	317 (18.12)	383 (16.77)	9 (6.77)	69 (15.79)	78 (13.68)
COPD	147 (27.48)	451 (25.79)	598 (26.18)	34 (25.56)	84 (19.22)	118 (20.7)
Myocardial infarction	149 (27.85)	311 (17.78)	460 (20.14)	42 (31.58)	75 (17.16)	117 (20.53)
Angina	239 (44.67)	560 (32.02)	799 (34.98)	61 (45.86)	146 (33.41)	207 (36.32)

Values are number (percentage); rEF reduced ejection fraction, pEF preserved ejection fraction; COPD chronic pulmonary disease. CKD stage 3 glomerular filtration rate < 60 mL/min/1.73 m^2^.

**Table 2:** Proportion of HFpEF and HFrEF phenotypes in the original dataset and in the two balanced datasets created with *SMOTE*.

n (%)	Original	Balance 1	Balance 2
Total size	2284	2140	3745
HFpEF class	1749 (76.58)	1070 (50)	2140 (42.86)
HFrEF class	535 (23.42)	1070 (50)	1605 (57.14)

HFpEF, heart failure preserved ejection fraction. HFrEF, heart failure reduced ejection fraction.

**Table 3:** Performance measures of the predictive models in the Testing Dataset.

		AUC	AUCpr	Accuracy	Sensitivity	Specificity	PPV	NPV	HFrEF Class (%) *
XGBoost	Full models								
	Original	0.70	0.45	0.80	0.53	0.88	0.57	0.86	24.11
	Smote 50-50	0.69	0.38	0.70	0.69	0.70	0.41	0.88	26.05
	Smote balanced	0.65	0.35	0.72	0.53	0.77	0.41	0.84	21.63
	Reduced models								
	Original	0.70	0.46	0.81	0.49	0.90	0.60	0.85	17.47
	Smote 50-50	0.68	0.38	0.72	0.61	0.76	0.44	0.86	25.05
	Smote balanced	0.66	0.36	0.71	0.53	0.76	0.40	0.84	19.04
RF	Full models								
	Original	0.70	0.51	0.83	0.46	0.95	0.72	0.85	4.23
	Smote 50-50	0.69	0.38	0.73	0.65	0.75	0.44	0.88	16.57
	Smote balanced	0.72	0.44	0.77	0.62	0.82	0.51	0.88	15.42
	Reduced models								
	Original	0.70	0.51	0.84	0.46	0.95	0.75	0.85	3.8
	Smote 50-50	0.70	0.38	0.73	0.65	0.75	0.44	0.88	14.38
	Smote balanced	0.72	0.44	0.78	0.62	0.83	0.52	0.88	12.55

AUC area under the curve; AUCpr area under precision recall curve; PPV positive predictive value; NPV negative predictive value, RF Random Forest. * Percent of HFrEF class identified.

**Table 4:** Demographics and chronic diseases of the Heart Failure population tested.

Variables	All Subjects (n = 79,057)	Primary Care (n = 26,376)
Demographics		
Male	36,539 (46.22)	10,082 (38.22)
Age, mean (SD)	77.75 (11.35)	80.88 (10.36)
Comorbidities		
Atrial fibrillation	31,277 (39.56)	6571 (24.91)
Anemia	30,132 (38.11)	9197 (34.87)
Diabetes	31,607 (39.98)	9998 (37.91)
Hypertension	66,181 (83.71)	21,048 (79.8)
Obesity	17,599 (22.26)	3757 (14.24)
Pulmonary HTN	842 (1.07)	260 (0.99)
CKD	15,469 (19.57)	2018 (7.65)
Valve disorders	13,061 (16.52)	1016 (3.85)
COPD	20,569 (26.02)	6647 (25.2)
Myocardial infarction	13,243 (16.75)	2038 (7.73)
Angina	24,655 (31.19)	4727 (17.92)

CKD Chronic kidney disease. COPD Chronic obstructive pulmonary disease.

## Data Availability

Not applicable.

## Supplementary Materials

The following are available online at https://www.mdpi.com/article/10.3390/bioengineering8060085/s1, Figure S1. Study population flow chart.
