# Supplementary figures and images for: Supervised Analysis for Phenotype Identification: The Case of Heart Failure Ejection Fraction Class
Source: Bioengineering (Basel). 2021 Jun 21;8(6):85. doi: 10.3390/bioengineering8060085 (PMC8233943; doi:10.3390/bioengineering8060085)

Supplementary  
Figure S1

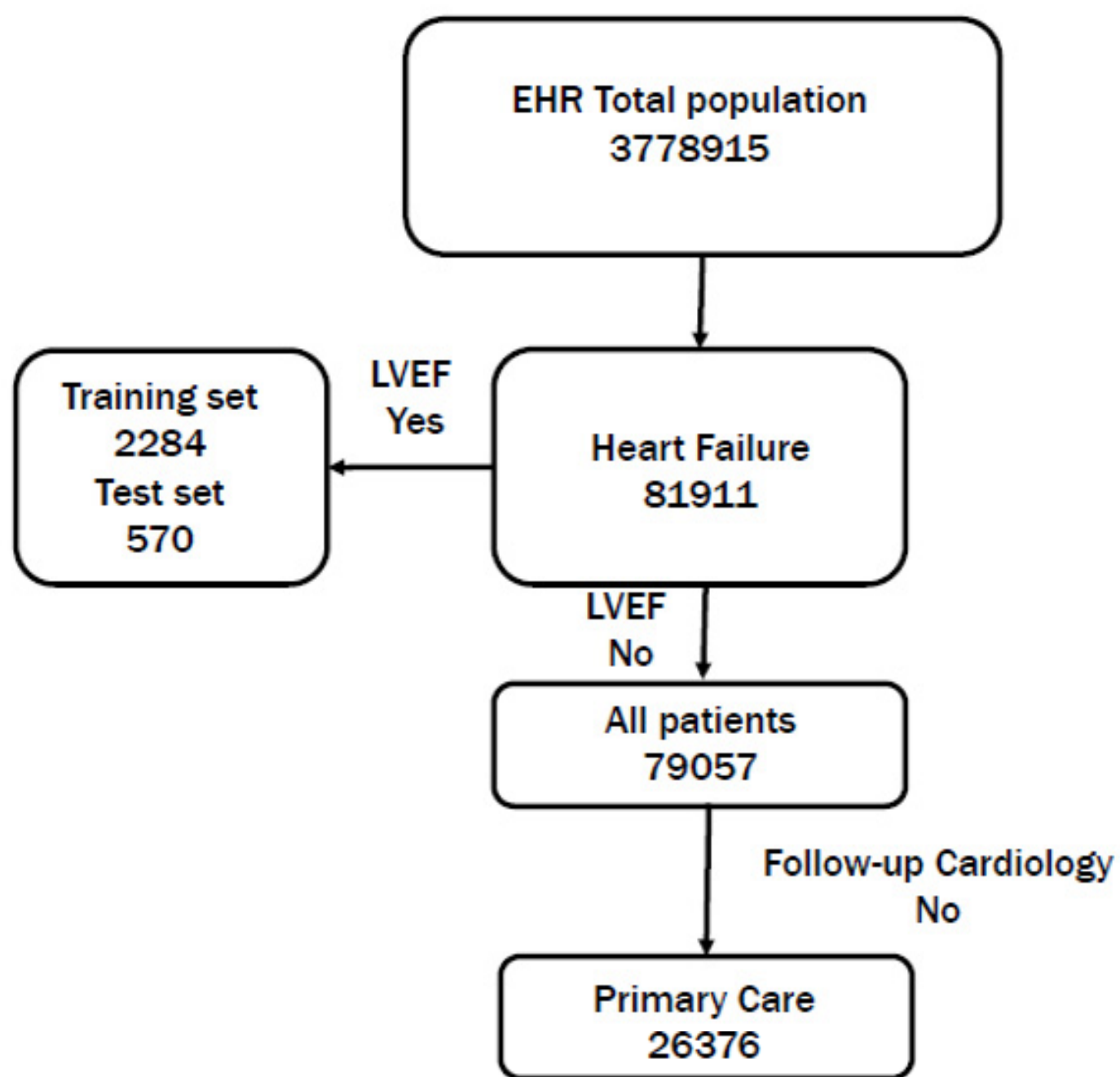

Supplement: Supplementary file 1 [file bioengineering-08-00085-s001.zip › bioengineering-1254557-SI.pdf]
